# Selecting the right medical student

**DOI:** 10.1186/1741-7015-11-245

**Published:** 2013-11-14

**Authors:** Sam Leinster

**Affiliations:** 1 Norwich Medical School, University of East Anglia, Norwich NR4 7TJ, UK

**Keywords:** Medical School Admission, Predictors of performance, Aptitude testing

## Abstract

Medical student selection is an important but difficult task. Three recent papers by McManus et al. in *BMC Medicine* have re-examined the role of tests of attainment of learning (A’ levels, GCSEs, SQA) and of aptitude (AH5, UKCAT), but on a much larger scale than previously attempted. They conclude that A’ levels are still the best predictor of future success at medical school and beyond. However, A’ levels account for only 65% of the variance in performance that is found. Therefore, more work is needed to establish relevant assessment of the other 35%.

Please see related research articles http://www.biomedcentral.com/1741-7015/11/242, http://www.biomedcentral.com/1741-7015/11/243 and http://www.biomedcentral.com/1741-7015/11/244.

## Background

The selection of students for medical school is challenging. Many more students apply than can be accommodated. At first sight this appears to be a good thing as it allows the best students to be accepted. The real problem lies in determining how ‘best’ should be defined. Is it the student who will perform best at medical school or the one who will perform best in a lifetime of medical practice? Are the two the same or are there different predictors for each? It is reasonable to define success at medical school in terms of examination results but how should success in medical practice be determined? The range of medical practice is immense and it is difficult to make meaningful comparisons between a doctor who spends his or her life as a well-respected General Practitioner serving a severely deprived inner-city area, a high profile Consultant who becomes President of a Royal College, or a Public Health doctor who champions a healthy living program that results in a fall in the incidence of Type 2 diabetes in adolescents.

The Royal College of Physicians and Surgeons of Canada identify six domains of competence that, together, make up the medical expert [1]:

• Communicator

• Collaborator

• Manager

• Health Advocate

• Scholar

• Professional

Doctors from whatever discipline can, in theory, be judged by the extent to which they display these competencies in a way that is relevant within their given scope of practice. The difficulty is the lack of a comprehensive and robust measure for most of these competences. Scholarship is correlated with knowledge and can, therefore, be reliably assessed. The other domains have a strong behavioral component, and it is difficult to assess the doctor’s performance within these domains in the workplace.

Traditionally, selection for medical school was based on measures of the student’s knowledge, in some cases supplemented by an interview that was often *ad hoc* and unstructured. An increasing awareness of the range of competencies that are needed for competent medical practice led to calls for more transparent and rigorous processes. In 1998, Powis [2] suggested a process that combined a threshold academic performance level, scores on a psychometric test and scores at a structured interview in order to reach a decision on admission. By 2013, the majority of UK medical schools were following this protocol but it is still not clear what each of the components contributes to the process. The majority of UK medical schools use the UK Clinical Aptitude Test (UKCAT) as the psychometric test [3]. UKCAT is a web-based test of mental aptitude administered in test centers throughout the world. There are five sections: Quantitative Reasoning, Abstract Reasoning, Decision Analysis, Verbal Reasoning and Situational Judgement Test [4]. Students are required to sit the test in the year in which they apply to medical school.

### Recent evidence underlying performance of selection criteria for medical students

Three recent papers from McManus *et al*. [5-7] have attempted to address this issue, as far as academic achievement and psychometric testing are concerned. In contrast to previous studies that have suffered from small sample sizes or short follow up (focusing on performance in medical school rather than performance as a doctor), these present studies have combined cohorts across years and from different medical schools in order to achieve a large sample. They have attempted to analyze postgraduate performance by reference to performance in the Membership of the Royal Colleges of Physicians UK (MRCP(UK)) examination and entry on to the General Medical Council (GMC) Specialist Register. Perhaps the most useful feature of their studies is that they have not only examined the predictive validity of the measures for performance in medical school of those admitted, but have estimated the utility of the measures as a selection tool by calculating the construct-level predictive ability. Briefly, construct-level predictive validity of a selection measure is the association between the construct assessed by the selection measure, the predictor, and the medical knowledge, skills and attitudes measured by later undergraduate and postgraduate examinations, the outcomes. Because this estimate applies to the whole population of applicants and not just to those admitted, it gives a truer picture of the value of a given tool for selection.

The first study [4] examined the construct predictive validity of selection measures in five cohort studies ranging from 1985 to 2006 and in one cross sectional study in 2007/2009. The study examined tests of academic attainment (A’ levels, General Certificate of Secondary Education (GCSE), Scottish Qualifications Authority) and aptitude (UKCAT). While all of the tests were predictive of performance in the first year of study, A’ level scores were the most predictive. The predictive power of the A’ level scores persisted at smaller but still significant levels into the postgraduate period. Aptitude tests added little incrementally to the A’ level score.

Overall, the A’ level score accounted for 65% of the observed variance in performance. The authors note that there is 35% of the variance that is unaccounted for. This reduces the effectiveness of the selection process but as yet it is not clear what factors give rise to this or how they could be assessed.

The second study [5] focused on the cross-sectional study which looked at the construct predictive validity of UKCAT, measures of academic attainment, and some demographic and sociological factors across three years (2006 through 2008) and within 12 UK medical schools. Because of time constraints, the study was limited to performance in the first academic year. Once again, prior educational attainment was the major predictor of performance and should continue to be used in selection decisions. UKCAT provided a small increment in validity, which the authors thought would be of value in actual admissions decisions when the data from educational attainment was incomplete. Interestingly, UKCAT showed greater predictive value in female students and mature students. The contextual variables showed some effect on performance, with male students performing less well than female and non-White students performing less well than White. Students from schools with a higher average performance at A’ level did less well than students from schools with a lower average once adjustment had been made for their personal academic attainment. The predictive value of the tests was the same irrespective of the medical school.

The third study [6] focused on the five cohort studies examining the correlations and path analyses between all stages of education, from GCSE and A’ level results through to postgraduate performance. The authors found strong correlations between performance at each stage of progression. This provides evidence for the concept of an academic backbone, that is, that those students who have previously performed well are likely to continue to do so, and supports the idea that measures of academic attainment such as A’ level results should continue to be an important component of selection for medical school.

### What are the implications of these studies for medical student selection?

The authors of the three papers have provided good evidence that A’ level results are a strong predictor of performance through medical school and into the early postgraduate years. However, they only account for 65% of the variance in performance and additional predictors are needed if selection is going to improve. Current alternative approaches, such as aptitude testing, appear to add little and are clearly not identifying the factors that make up the missing 35%.

The data do not include interview scores and it is not clear whether these would have improved the prediction. For example, in a separate study the use of structured interviews in Newcastle, New South Wales, provided a better prediction of failure to complete the course than academic achievement [8]. More recently, the Multiple Mini-interview (MMI) has been widely adopted. This has been shown to have good predictive validity for performance in the first year in a UK medical school [9] and for postgraduate performance in Canada [10].

It is not clear to what extent measurements of academic attainment are measuring the underlying level of intellectual ability, the importance of prior knowledge or factors such as conscientiousness and perseverance. The fact that the predictive value of A’ levels in students from schools with a high average achievement is less than it is in students from schools with a low average achievement [5] suggests that more than just the factual content of the A’ level is involved.

The weakness of all of the studies exploring factors predicting success in a medical career is the definition of that success. In all of the studies considered, success is defined in terms of performance in examinations. The recent McManus papers do include entry to the Specialist Register as a criterion for success, but as that is dependent on passing the relevant postgraduate examinations, it is not an entirely independent variable. It is perhaps unsurprising that proven ability to pass examinations should predict the ability to pass further examinations. There are further limitations arising from the focus in the postgraduate period on doctors choosing to sit the MRCP (UK). It is an unproven assumption that their behavior is an accurate reflection of doctors choosing other specialties. Factors other than ability to pass examinations may be important in other disciplines.

It is, in any case, by no means certain that examination performance is an accurate reflection of true clinical performance. At best, the examination occurs at the ‘shows how’ level of Miller’s triangle (a hierarchy of skills competency from ‘knows what’ through ‘knows how’, ‘shows how’ and ‘does’ to ‘mastery’ [11]). Attempts to get closer to the ‘does’ level of actual practice have given rise to attempts at workplace based or in-training assessments but even these, as currently constructed, may not give an accurate reflection of the doctor’s abilities [12]. Measuring the ultimate level of ‘mastery’ is even more of a challenge. Until we develop methods that allow meaningful measurement of clinical performance we will be forced to continue using surrogate measures which may not reflect the true situation. This affects decisions not only about selection processes but also about educational delivery.

## Conclusions

Despite these reservations, these papers are a useful contribution to the debate on medical student selection. The size of the sample and the variety of the cohorts suggest that the results may be generalizable. The thoroughness and attention to detail in the analysis is an example of good practice and lends authority to the conclusions. On this evidence, assessment of educational attainment will always be a necessary, but not sufficient, part of the selection process. Further studies are needed to determine what the other components should be.

## Abbreviations

A’ level: General certificate of secondary education advanced level; GCSE: General certificate of secondary education ordinary level; MRCP(UK): Membership of the Royal College of Physicians (UK); SQA: Scottish Qualifications Authority; UKCAT: United Kingdom Clinical Aptitude Test.

## Competing interests

The author declares that he has no competing interests.

## Authors’ information

SL is Emeritus Professor of Medical Education at the University of East Anglia. He was the Inaugural Dean of Norwich Medical School and was previously Director of Medical Studies at the University of Liverpool. His clinical practice was in General Surgery with a special interest in Surgical Oncology.
